# Paper-Based Screen-Printed Ionic-Liquid/Graphene Electrode Integrated with Prussian Blue/MXene Nanocomposites Enabled Electrochemical Detection for Glucose Sensing

**DOI:** 10.3390/bios12100852

**Published:** 2022-10-09

**Authors:** Wisanu Niamsi, Nutcha Larpant, Pramod K. Kalambate, Vitsarut Primpray, Chanpen Karuwan, Nadnudda Rodthongkum, Wanida Laiwattanapaisal

**Affiliations:** 1 Graduate Program in Clinical Biochemistry and Molecular Medicine, Faculty of Allied Health Sciences, Chulalongkorn University, Bangkok 10330, Thailand; 2 Biosensors and Bioanalytical Technology for Cells and Innovative Testing Device Research Unit, Department of Clinical Chemistry, Faculty of Allied Health Sciences, Chulalongkorn University, Bangkok 10330, Thailand; 3 Graphene Sensor Laboratory (GPL), Graphene and Printed Electronics for Dual-Use Applications Research Division (GPERD), National Security and Dual-Use Technology Center (NSD), National Science and Technology Development Agency (NSTDA), Phahonyothin Road, Khlong Nueng, Khlong Luang, Pathum Thani 12120, Thailand; 4 Metallurgy and Materials Science Research Institute, Chulalongkorn University, Bangkok 10330, Thailand; 5 Center of Excellence in Responsive Wearable Materials, Chulalongkorn University, Bangkok 10330, Thailand

**Keywords:** glucose biosensor, composite nanomaterials, MXene, prussian blue, glucose oxidase, paper based analytical device, real sample analysis

## Abstract

As glucose biosensors play an important role in glycemic control, which can prevent the diabetic complications, the development of a glucose sensing platform is still in needed. Herein, the first proposal on the in-house fabricated paper-based screen-printed ionic liquid/graphene electrode (SPIL-GE) modified with MXene (Ti_3_C_2_T_x_), prussian blue (PB), glucose oxidase (GOx), and Nafion is reported. The concentration of PB/Ti_3_C_2_T_x_ was optimized and the optimal detection potential of PB/Ti_3_C_2_T_x_/GOx/Nafion/SPIL-GE is −0.05 V. The performance of PB/Ti_3_C_2_T_x_/GOx/Nafion modified SPIL-GE was characterized by cyclic voltammetry and chronoamperometry technique. This paper-based platform integrated with nanomaterial composites were realized for glucose in the range of 0.0–15.0 mM with the correlation coefficient R^2^ = 0.9937. The limit of detection method and limit of quantification were 24.5 μM and 81.7 μM, respectively. In the method comparison, this PB/Ti_3_C_2_T_x_/GOx/Nafion/SPIL-GE exhibits a good correlation with the reference hexokinase method. This novel glucose sensing platform can potentially be used for the good practice to enhance the sensitivity and open the opportunity to develop paper-based electroanalytical devices.

## 1. Introduction

Diabetes mellitus, a leading non-communicable disease-related health issue, is a risk factor for a variety of vascular illnesses [1]. By 2045, the predicted worldwide prevalence will reach 738.2 million cases [2]. This tremendous number led to the long-term health effects, mortality rate, and economic and financial burden. Regards to Diabetes self-management education and support from Centers for disease Control and prevention (CDC), the discipline in glycemic control can prevent and delay these serious health complications including diabetic retinopathy, peripheral neuropathy, chronic kidney disease, cerebrovascular disease, and heart disease [3]. As the vital demand of more than 420 million people with diabetes globally for the blood glucose control and monitoring result to rapidly growing in the glucose biosensor market [4]. This statistic emphasized the continuous development of glucose sensing along with good practice for effective diabetic management. The inspiring aspects of improving the performance of glucose sensor focus on accuracy, precision, sensitivity, stability, ease of use, miniaturization, and connectivity to smart devices [5].

Non-enzymatic glucose detection has been greatly enhanced by ongoing glucose electrochemical sensing research; nonetheless, poor selectivity and the optimum working out of physical pH remain obstacles to their widespread use in clinical diagnosis and commercially available glucose sensors [5]. The glucose oxidase enzyme has been widely utilized in glucose biosensors despite still being systematically and critically investigated after half a century due to its exceptional features, including high stability in varying pH and temperature, and high specificity to the glucose substrate [6,7,8]. Due to the fact that GOx catalyzes glucose to generate H_2_O_2_, this byproduct may be detected by highly sensitive electrochemical techniques and is proportional to the amount of glucose in the blood [7].

Many efforts on development of non-invasive glucose sensing in order to painless detection however the sensitivity, accuracy, and stable of this type of sensing still be the obstacles for glucose measurement that reflect the accurate health status for clinical diagnosis [9]. For these reasons, blood glucose is still important for monitoring when compared to other biological sample matrices, e.g., sweat and saliva.

In addition to sample matrix aspect, the materials used in glucose sensor development have been extensively studied in many scopes to enhance the performance of sensor. Nanostructures provide many benefits, including a higher surface area to volume ratio, greater enzyme loading and immobilization capacity, enhanced electron transfer rate, catalytic activity, and conductivity [10]. Different types of nanomaterials are also extensively studied and applied to enhance the electrochemical glucose sensing performance. Prussian blue (PB) is one of the nanomaterials that have been popularly utilized as an artificial peroxidase [11]. The low potential transducer of hydrogen peroxide is the outstanding property of PB, which is superior to other known systems. This strategy has been applied in glucose determination to prevent the disturbed signal from interference species at high potential [12]. In addition, the combination between Prussian blue and other materials is one of the strategies applied to meet the superior features of the sensors in biomedical application. Among various type of materials, Ti_3_C_2_T_x_ MXene have emerged as two-dimensional (2D) layered materials that gaining lots of attractions in the area of sensor application [13]. MXenes are made of carbides, nitrides, or carbonitrides of transition metals. Their inherited metallic-like conductivity from their parent MAX precursors and excellent hydrophilicity offered by the surface terminating moieties [14]. Ti_3_C_2_T_x_ MXenes possess various distinctive properties, for instance, sizable active surface area, good electrocatalysis, and superior electrical conductivity, which make these materials highly promising for developing sensitive electrochemical sensors and biosensors for numerous analytes [15,16]. As an advantage, Ti_3_C_2_T_x_ MXene nanosheets have been implemented in improving the performance of glucose sensors [17]. Graphene also accounts for two-dimensional layered materials which attract lots of interest due to their advantages such as electrical, thermal, mechanical, and optical properties [18]. Due to the excellence conductivity, high surface area, catalytic activity toward H_2_O_2_ of graphene, it is widely used in the glucose sensor field [19]. Apart from nanomaterials, the paper-based analytical device (PAD) is a particularly attractive alternative for clinical diagnosis and setting. Due to the low cost, small volume of reagent, miniaturization, ease of manipulate, and integration with other assays [20], PAD can be an ideal proposed platform for practical electrochemical glucose sensing, especially for single-use blood glucose detection.

According to the pioneer work on screen printing of ionic liquid/graphene on poly-ethylene terephthalate substrates, the screen-printed ionic liquid/graphene electrode can enhance the sensitivity of ferri/ferro cyanide (Fe (CN)_6_)^3−/4−^, dopamine and hydroquinone detection when compared to screen-printed carbon and graphene electrodes because the ionic liquids help the dispersion of graphene which increases the conductivity [21]. However, there are no reports on applying this prototype paper based screen-printed ionic liquid electrode for glucose sensing.

Herein, we are first proposed the in-house developed paper-based screen-printed ionic liquid/graphene electrode (SPIL-GE) modified with Ti_3_C_2_T_x_, PB, GOx, and Nafion. This proposed modified SPIL-GE was implemented to monitor glucose levels in real plasma samples. Ti_3_C_2_T_x_ was modified to increase the enzyme loading capacity on the electrode surface [22] incorporated with the catalytic activity of PB toward H_2_O_2_ [23]. This modification to paper-based SPIL-GE may increase sensitivity toward glucose detection. In addition, Nafion was also utilized to avoid the negatively charged interferences in sample matrices such as ascorbic acid, uric acid, and other proteins [24,25]. Firstly, GOx was loaded on MXene/PB layers to catalyzed glucose in the sample matrix to gluconolactone and hydrogen peroxide. Secondly, hydrogen peroxide, a by-product from the first reaction, can be catalyzed by PB at low potential. The response signal of the reaction can be monitored by amperometric measurement. In this study, Whatman No.1 was applied to assist the delivery of sample by capillary force to the working electrode area which allows portable measurement. This paper-based SPIL-GE was characterized and tested with real sample matrices. This study may enhance the knowledge on the modification of paper-based electrodes. This integrated paper-based sensor for glucose sensing may be wirelessly connected to a smart phone for monitoring and providing personal health information to medical staff, which could improve the efficacy of diabetes management in remote areas and be useful for portable in-field analysis [26,27].

## 2. Materials and Methods

### 2.1. Chemicals and Materials

Glucose oxidase from *Aspergillus niger* (>125U), titanium aluminium carbide (Ti_3_AlC_2_) powder, potassium ferricyanide (K_3_[Fe(CN)_6_]), potassium ferrocyanide trihydrate (K_4_[Fe(CN)_6_]·3H_2_O), iron (III) chloride (FeCl_3_), potassium chloride (KCl), hydrochloric acid (HCl), lithium fluoride (LiF), D-(+)-glucose, Nafion 117 (~5%), sodium di-hydrogen phosphate monohydrate (NaH_2_PO_4_·H_2_O), di-sodium hydrogen phosphate dihydrate (Na_2_HPO_4_·2H_2_O), sodium chloride (NaCl), ascorbic acid, uric acid, and hemoglobin were obtained from Sigma-Aldrich (St. Louis, MO, USA). Whatman No. 1 filter paper was purchased from Whatman International Ltd. (Maidstone, England) and A4 230-g card paper was purchased in Bangkok, Thailand. In this work, total amounts of 0.2 mM stock solution of glucose were prepared by dissolving D-(+)-glucose in 0.1 M PBS (pH 7.4). The solution of 10 mg/mL glucose oxidase enzyme was prepared by dissolving it in 50 mM sodium acetate (pH 5.1). The deionized water used in this study was obtained in the laboratory by using ELGA LabWater purification system (0.067 μS/cm conductivity, resistivity of 18 MΩ.cm at 25 °C).

### 2.2. Apparatus

All electrochemical characterization was carried out using a portable PalmSens4 (PalmSens, Houten, The Netherlands) (Figure S1a). For the electrochemical detection of blood glucose, chronoamperometry was tested by using USB-C Sensit Smart (PalmSens, Houten, The Netherlands) with PStouch app for Android smart phone as showed in Figure S1b. A CO_2_ laser cutting machine (60 Watt.) (Cnmanlaser, model MAN-6090, Qingdao, China) was purchased from the MIT group, Thailand. Field emission scanning electron microscope (FESEM) analysis were performed at the National Science and Technology Development Agency, Thailand. HITACHI SU8030 FESEM (Tokyo, Japan) were used to study the morphology of the modified electrode.

The electrochemical properties of PB/Ti_3_C_2_T_x_/GOx/Nafion on SPIL-GE were characterized by cyclic voltammetry (CV) with 5 mM ferri/ferrocyanide in 0.1 M KCl solution. The chronoamperometry technique was used for characterization to find the optimal concentration of modified nanomaterial and selection of optimal detection potential conditions for quantitative determination of glucose.

### 2.3. Fabrication of Screen-Printed IL/Graphene Electrodes (SPIL-GEs) on Paper Based

**Figure 1:**
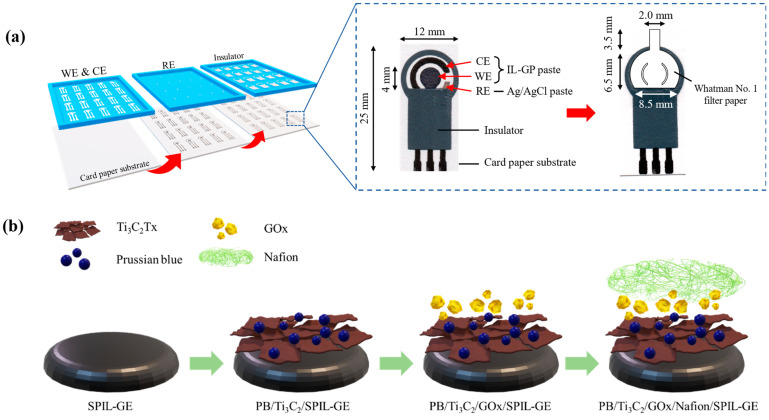
The fabrication process of paper-based SPIL-GEs glucose sensor. (**a**) fabrication steps of SPIL-GEs on paper based using IL-GP paste as WE and CE, Ag/AgCl paste as RE, and insulator paste, respectively; inset, details of each SPIL-GE (left); design and size of Whatman No.1 filter paper applied on the SPIL-GE (right), (**b**) modification steps of PB/Ti_3_C_2_T_x_/GOx/Nafion on paper-based SPIL-GE.

To Fabricated of SPIL-GEs on paper based, The commercial ionic liquid graphene ink (IL-GP ink) was composed of a conductive carbon paste, exfoliated graphene powder in poly(3,4-ethylenedioxythiophene) polystyrene sulfonate (PEDOT/PSS) solution and 3-methyl-1-propylpyridinium bis(trifluoromethyl sulfonyl)imide (PMPlm) as a weight ratio of 100:2.5:1 [21]. In Figure 1a, the IL-GP ink was screened through the pattern stencil mesh on the A4 card paper with a thickness of 230 g for the fabrication of the working electrode (WE with a diameter of 4 mm.) and counter electrode (CE) by using a screen printer (DEK version 03ix). Next, silver/silver chloride paste (Ag/AgCl) and insulator paste was also screened on the same electrode for the fabrication of a references electrode (RE) and detection area, respectively. Each screened layer was dried to remove solvent by baking at 60 °C about 5 min and storage at room temperature for this application.

### 2.4. Preparation of Modified PB/Ti_3_C_2_T_x_/GOx/Nafion on Paper-Based Screen-Printed Ionic Liquid/Graphene Electrode (SPIL-GE) Glucose Sensor

To prepared Ti_3_C_2_T_x_ flakes, Ti_3_C_2_T_x_ were synthesized by the modified minimally intensive layer delamination (MILD) method using in situ HF formation from HCl and LiF to prepare both etchant and intercalation in exfoliation process which is less hazardous and provides an easier step than using HF where the flakes are isolated by manual shaking without further intercalation [28,29]. The in situ HF etchant was prepared by adding 1.0 g of LiF to 20 mL of 9 M HCl under continuous stirring to acquire homogeneous mixture. Next, a total of 1.0 g of Ti_3_AlC_2_ (MAX phase) powder was then slowly added to the etchant and allowed the reaction to run under continuous stirring at 50 °C for 50 h. The resulting mixture was centrifuged and washed with deionized water to remove the acidic supernatant (Figure S2a) and repeat washing cycles until the pH of supernatant reached to pH 5–6. After being washed, the black Ti_3_C_2_T_x_ layer and a grey layer of Ti_3_AlC_2_/Ti_3_C_2_T_x_ mixture were settle at the bottom of the centrifuge tube as showed in Figure S2b [29]. The Ti_3_C_2_T_x_ clay was transferred to a glass petri dish and dried at 65 °C for 12 h to obtain the Ti_3_C_2_T_x_ film (Figure S2c). Ti_3_C_2_T_x_ film was kept under argon gas in a sealed aluminum foil bag for further use.

For PB/Ti_3_C_2_T_x_, the composite was synthesized via the in situ reduction process as described in previous studies [30]. The PB nanoparticles were produced by reduction of the Fe(III) and Fe(CN)_6_^3−^ mixture with Ti_3_C_2_T_x_, where Fe(III) was reduced to Fe(II) and Fe(CN)_6_^3–^ was reduced to Fe(CN)_6_^4−^. To synthesize, the total amount of 13.5 mg of FeCl_3_ and 16.5 mg of K_3_[Fe(CN)_6_] were dissolved in 10 mL of 0.1 M KCl (pH 1.5) for 30 min. A total 10 mL of 2 mg/mL Ti_3_C_2_T_x_ suspension was slowly added into the mixture solution of FeCl_3_ and K_3_[Fe(CN)_6_]. After continuous stirring for 30 min, the precipitated PB/Ti_3_C_2_T_x_ were filtrated by vacuum assisted filtration and washed with deionized water for 5 times. The resulting PB/Ti_3_C_2_T_x_ nanocomposites were obtained after being dried at 60 °C for 12 h.

### 2.5. Fabrication of the Paper-Based Glucose Sensor

To fabricate the devices, paper-based SPIL-GEs were electrochemically cleaned to remove contaminants on electrode surfaces [21]. Briefly, 80 µL of 0.1 M PBS solution (pH 7.4) was dropped onto the electrode surface and applied the voltage between−0.3 and +0.6 at a scan rate of 50 mV/s for 5 scans by using the cyclic voltammetry (CV) technique. After being cleaned, paper-based SPIL-GEs were carefully washed with deionized water and dried at 60 °C for 15 min. Before applying PB/Ti_3_C_2_T_x_ nanocomposites on the electrode surface, 3 mg/mL PB/Ti_3_C_2_T_x_ was dispersed in deionized water and the suspended solution was sonicated for 90 min to acquire a homogeneous mixture. In Figure 1b, total 8 μL of PB/Ti_3_C_2_T_x_ mixture was then dropped cast on paper-based SPIL-GE and dried at 60 °C for 30 min. After the electrode was cooled for 10 min, 6 μL of 10 mg/mL GOx in sodium acetate (pH 5.1) was dropped and dried at 25 °C for 2 h. Subsequently, 2 μL of 0.5% Nafion was dropped at room temperature for 1 h. Finally, the designed Whatman No.1 was cut by using CO_2_ laser cutting machine and applied on the working electrode area with transparent adhesive tape to assist the delivery of sample by capillary force, as showed in Figure 1a. The modified paper-based microfluidic glucose sensor was store in sealed aluminum foil bag at 4 °C before use.

### 2.6. Real Sample Analysis and Interference Studies

The paper-based SPIL-GE device was used to determine blood glucose level by using human blood plasma (n = 10) and compared with enzymatic glucose assay (Hexokinase) using automated analyzer ARCHITECT ci4100 (Abbott, IL, USA). The concentration of ascorbic acid (0.1 mM), uric acid (0.1 mM), and hemoglobin (0.06 mM) in PBS were used to study the effects of the main interferences in human blood plasma.

To verify the electrochemical method for real sample analysis, the prepared paper-based PB/Ti_3_C_2_T_x_/GOx/Nafion/SPIL-GE was used to measure blood glucose concentration. In this work, separated blood plasma was carefully collected from sodium fluoride (NaF) containing tube after drawing the whole blood sample from volunteers and centrifuged at 3000 rpm for 10 min. The human blood plasma was then determined to have glucose concentration in plasma using paper-based PB/Ti_3_C_2_T_x_/GOx/Nafion/SPIL-GE that was connected to Sensit Smart. In the process of real sample assay, the sample matrix is one of the crucial factors that effects the obtained signal because it contains lots of interference, including ascorbic acid, and uric acid. To minimize the sample matrix effects, we diluted the plasma sample with PBS at a ratio of 1 to 3. Furthermore, the Nafion was utilized to trap the negatively charged interference species that may affect the real sample of plasma glucose measurements.

## 3. Results and Discussions

### 3.1. Electrochemical Characterization of the Modified Electrodes

**Figure 2:**
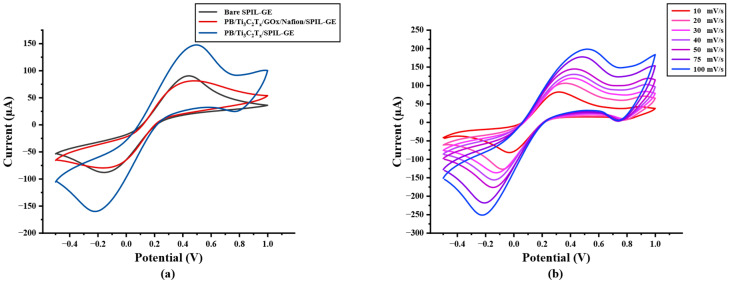

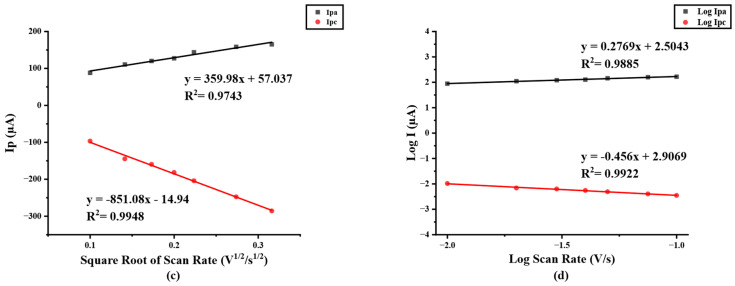
(**a**) Cyclic voltammetry of bare SPIL-GE, modified PB/Ti_3_C_2_T_x_/SPIL-GE, and PB/Ti_3_C_2_T_x_/Gox/Nafion/SPIL-GE with 5 mM [Fe(CN)_6_]^3−/4−^ in 0.1 M KCl, (**b**) Cyclic voltammetry of PB/Ti_3_C_2_T_x_/SPIL-GE at various scan rates of 10–100 mV/s with 5 mM [Fe(CN)_6_]^3−/4−^ in 0.1 M KCl, (**c**) Plots of anodic and cathodic peak currents and square root of scan rate for PB/Ti_3_C_2_T_x_/SPIL-GE and (**d**) Logarithm plots of anodic and cathodic peak currents and logarithm of scan rate.

The electrochemical behavior of modified electrodes in this work was investigated in the presence of a 5 mM redox probe [Fe(CN)_6_]^3−/4−^ in 0.1 M KCl by cyclic voltammetry (CV) at a potential range between −0.5 to +1.0 V/s with a scan rate of 50 mV/s. As shown in Figure 2a, after the electrode was modified, the redox peak current response of modified PB/Ti_3_C_2_T_x_ nanocomposites on SPIL-GE was distinctly enhanced due to the excellent electroactivity of PB nanoparticles and the multilayer of Ti_3_C_2_T_x_ sheets. Furthermore, the redox peak currents of PB/Ti_3_C_2_T_x_/GOx/Nafion/SPIL-GEs were also studied. Immobilized GOx and Nafion on PB/Ti_3_C_2_T_x_ showed a significant decrease in the redox peak currents, which may be affected by the fact that GOx is a protein and Nafion also has an insulation property that may block the electron transfer between the redox probe and electrode surfaces [25,31]. To characterize modified PB/Ti_3_C_2_T_x_ on SPIL-GEs, the electrochemical behavior property was investigated. The ∆Ep values obtained for bare SPIL-GE, PB/Ti_3_C_2_T_x_/SPIL-GE, and PB/Ti_3_C_2_T_x_/GOx/Nafion/SPIL-GE were 279, 239, and 299 mV, respectively. When the SPIL-GE was modified with PB/Ti_3_C_2_T_x_, the ∆Ep value decreased due to the high surface area and excellent electrical conductivity of MXene. However, when PB/Ti_3_C_2_T_x_/SPIL-GE was modified with GOx/Nafion, the ∆Ep value again increased due to the insulating nature of GOx and nafion. Since the behavior of PB does produce a redox peak signal, PB/Ti_3_C_2_T_x_ modified electrode have been studied with 0.1 M KCl to verify the redox process of PB. In Figure S3, CV of PB/Ti_3_C_2_T_x_/SPIL-GE showed the resulting of clear redox peak current compare with bare-SPIL-GE and Ti_3_C_2_T_x_ modified SPIL-GE. Figure 2b, the cyclic voltammograms (CVs) of PB/Ti_3_C_2_T_x_ modified SPIL-GEs (PB/Ti_3_C_2_T_x_/SPIL-GEs) at different scan rates in range of 10–100 mV/s with 5 mM [Fe(CN)_6_]^3−/4−^ in 0.1 M KCl solution showed a pair of redox peaks which were the oxidation and reduction peak currents. To investigate the electrochemical behaviors, Figure 2c showed the relationship between the square root of scan rate and redox peak currents. The electroactive properties of the oxidation peak currents and reduction peak currents were linearly related to the square root of the scan rate between 10 to 100 mV/s with the linear correlation (R^2^) of 0.9743 and 0.9948, respectively. Indicating the diffusion control behavior.

In addition, A plot of logarithm of redox peak currents vs. logarithm of scan rate were investigated. Figure 2d shows a good linear correlation with the slope of 0.456 which indicating that the reduction reaction has a diffusion control behavior.

### 3.2. The Topographical Analysis of Ti_3_C_2_T_x_ and PB/Ti_3_C_2_T_x_

**Figure 3:**
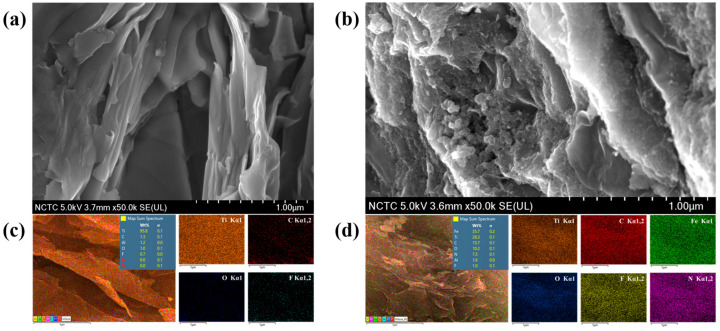
SEM micrographs of Ti_3_C_2_T_x_ at 50,000× (**a**), and PB/Ti_3_C_2_T_x_ at 50,000× (**b**). EDS elemental mapping analysis of Ti_3_C_2_T_x_ (**c**), and PB/Ti_3_C_2_T_x_ (**d**).

FESEM was employed to investigate the morphological analysis of Ti_3_C_2_T_x_ and PB/Ti_3_C_2_T_x_. The micrographs of Ti_3_C_2_T_x_ and PB/Ti_3_C_2_T_x_ were recorded under the accelerating voltage of 5.0 kV and magnifications of 50,000× as showed in Figure 3a,b.

The elemental mapping analysis was carried out using EDS analysis to study elemental distribution in Ti_3_C_2_T_x_ and PB/Ti_3_C_2_T_x_ nanocomposites. Figure 3c show distinctly demonstrates of the existence and distribution of Ti, C, O, and F elements in Ti_3_C_2_T_x_ after delamination and further confirmed the elemental analysis of Fe and N elements in PB/Ti_3_C_2_T_x_ nanocomposites as showed in Figure 3d.

### 3.3. Selection the Optimal Concentration of PB/Ti_3_C_2_T_x_ Modified on Paper-Based SPIL-GE

**Figure 4:**
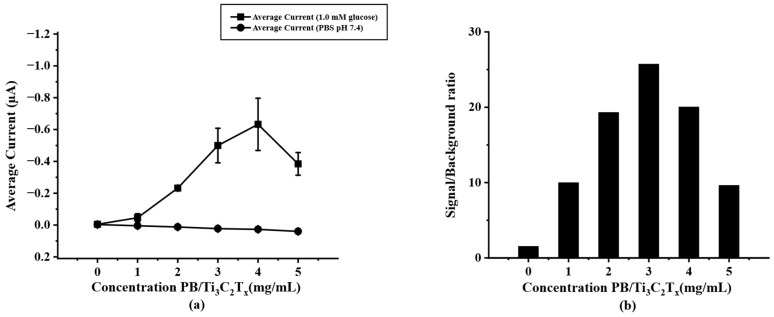
(**a**) current response of PB/Ti_3_C_2_T_x_/Gox/Nafion/SPIL-GE at different concentrations of PB/Ti_3_C_2_T_x_ nanocomposites using chronoamperometry (applied potential at 0 V vs. Ag/AgCl) in 1.0 mM glucose, (**b**) S/B ratio at different concentrations of PB/Ti_3_C_2_T_x_ nanocomposites according to the results in figure (**a**).

To optimize the modified PB/Ti_3_C_2_T_x_ on paper-based SPIL-GE, varying concentrations of PB/Ti_3_C_2_T_x_ nanocomposites were prepared as mentioned above. (Section 2.5) To select the optimal concentration of PB/Ti_3_C_2_T_x_ for further experiments, chronoamperometry was selected to test the difference concentration of modified PB/Ti_3_C_2_T_x_/SPIL-GE with 1.0 mM glucose in 0.1 M PBS (pH 7.4). As shown in Figure 4a, the result showed that, the more concentration of PB/Ti_3_C_2_T_x_ on SPIL-GE the current response was increases until the concentration reaches to 4 mg/mL. However, the background and standard deviation (error bar) at a concentration of 4 mg/mL PB/Ti_3_C_2_T_x_ were also increased. While the concentration of 3 mg/mL PB/Ti_3_C_2_T_x_ gives the highest signal/background ratio as showed in Figure 4b. Therefore, the concentration of 3 mg/mL was chosen as the optimal concentration of PB/Ti_3_C_2_T_x_ for modified SPIL-GE due to the lower in standard deviation and the highest current of signal/background ratio.

### 3.4. Selection the Optimal Detection Potential of PB/Ti_3_C_2_T_x_/Gox/Nafion/SPIL-GE

**Figure 5:**
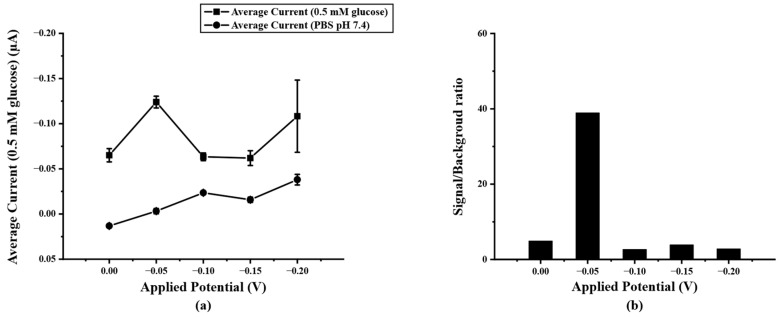
(**a**) current response of PB/Ti_3_C_2_T_x_/Gox/Nafion/SPIL-GE at different applied potential (0.0~−0.20 V vs. Ag/AgCl) in 0.5 mM glucose, (**b**) S/B ratio at different applied potential according to the results in figure (**a**).

After optimizing the concentration of PB/Ti_3_C_2_T_x_ on SPIL-GE with chronoamperometry, the detection potential for determining glucose concentration was also optimized. To select the optimal detection potential for acquiring the lowest detection limit, the highest current response with the lowest background current was considered. Therefore, the final concentration of 0.5 mM glucose in 0.1 M PBS pH 7.4 was added and measured at fixed different potentials of 0.00, −0.05, −0.10, −0.15, and −0.20 V (vs Ag/AgCl) compared with 0.1 M PBS pH 7.4 as the background current response. In Figure 5a, it can be seen that the applied potential of −0.05V (vs. Ag/AgCl) shows a high current response than the rest. Therefore, the −0.05 V was chosen due to the lowest applied potential to avoid interference from applying the high potential and give the highest signal/background ratio as shown in Figure 5b.

### 3.5. Analytical Performance of Glucose Determination on PB/Ti_3_C_2_T_x_/Gox/Nafion/SPIL-GE

**Figure 6:**
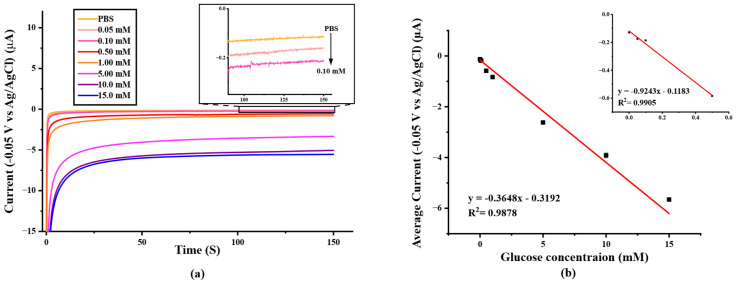
(**a**) Chronoamperometric response to the increasing of glucose concentration from 0–15 mM in 0.1 M PBS pH 7.4 (applied potential at −0.05 V vs. Ag/AgCl), (**b**) A calibration curve with the linear range at 0.0–15.0 mM glucose in 0.1 M PBS (pH 7.4) (R^2^ = 0.9878). Inset; a calibration curve with the linear range at 0.0–0.5 mM glucose. (R^2^ = 0.9937)**.**

To determine the analytical performance of the paper-based SPIL-GE biosensor, the different concentrations of glucose were prepared and measured by using chronoamperometry with an applied detection potential of −0.05 V (Figure 6a) and the determination result was recorded and constructed as a calibration curve by plotting between average amperometry current responses (t = 100–150 s) and the prepared standard glucose concentration in the range of 0.0–15.0 mM as shown in Figure 6b. The linear range of glucose determinations are 0.0–0.50 and 0.0–15.0 mM with the correlation coefficient R^2^ = 0.9937 (LOD = 24.5 μM) and 0.9878 (LOD = 62.1 μM), respectively. These results indicate that, the paper-based PB/Ti_3_C_2_T_x_/Gox/Nafion/SPIL-GE showed an excellent electrochemical response for glucose determination.

### 3.6. Real Sample Analysis of the Paper-Based PB/Ti_3_C_2_T_x_/Gox/Nafion/SPIL-GE and Interferences Study

**Figure 7:**
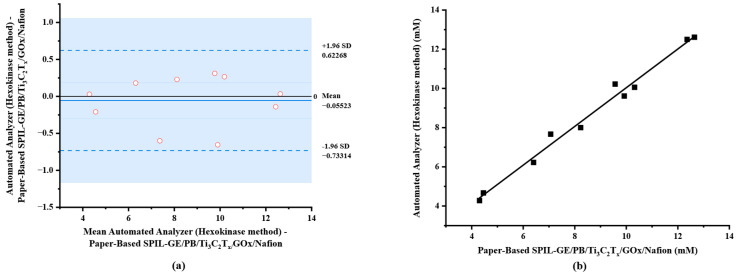
Comparison of electrochemical method using paper-based PB/Ti_3_C_2_T_x_/GOx/Nafion/SPIL-GE and automated analyzer (Hexokinase method). (**a**) A Bland–Altman bias plot; (**b**) correlation plot.

In this study, blood samples with spiked glucose at different concentrations in the range of 4.28–12.61 mM (n = 10) were analyzed using diluted procedure as mentioned above. The results were compared with the reference hexokinase/glucose-6-phosphate dehydrogenase (G6PD) method using automated analyzer (Architect ci4100). As shown in Table 1, the determination results of glucose using both electrochemical method on paper-based PB/Ti_3_C_2_T_x_/Gox/Nafion/SPIL-GE and automated analyzer (Hexokinase method) were compared. The result demonstrated that no bias for measurement of glucose in any samples was obtained, since the difference between the two methods fell within the ±1.96 standard deviation (SD) as shown in Figure 7a.

A scatter plot of the results obtained from both methods was constructed in which a correlation coefficient of 0.9846 (Figure 7b) was obtained. To compare two population means with 95% confidence interval, the degree of freedom of 9 and the critical t-value (t_0.05, 9_) was calculated to be ±2.262. Paired sample statistical analysis revealed that the experimental value of |t| was 1.022, a significantly lower than the critical t-value so the null hypothesis assumed no differences in means between the results of the two methods. This implies that our proposed electrochemical paper-based device can be used in the application of glucose analysis with the portable electrochemical device.

In addition, this research is the first to use screen-printed ionic liquid electrode on a paper substrate. The development is further studies of previous work on disposable screen-printed ionic liquid/graphene on polyethylene (PET) substrates [21]. A procedure used to prepare PB/Ti_3_C_2_T_x_ nanocomposites for easy electrode surface modification based on the process of reacting reeducation between Ti_3_C_2_T_x_ and Prussian Blue precursors [30], which is a convenient procedure compared to other methods of nanocomposite preparation [32,33,34]. To compare the electrochemical performance of the proposed sensor, the limit of detection and the analytical range were evaluated and compared with the sensors for glucose monitoring that have been reported in the past, and the results are described in Table 2. It is clear that the newly created sensor has a wider detection range and more sensitive detection capabilities than the majority of other sensors. As a result, the newly designed sensor demonstrates very good detection capabilities for glucose across a wider linear range. Therefore, there is a possibility that the electrochemical sensor might be used for the sensitive detection of glucose.

To investigate the selectivity of the proposed electrode toward glucose detection, the potential interferences in blood plasma including uric acid, ascorbic acid, and hemoglobin at their significant interfering concentration were added into 5 mM of glucose. The amperometric measurements were carried out and the % recoveries were reported as shown in Table 3. According to the obtained % recoveries, which are higher than 90%, these results verify that this designed electrode system is very selective for glucose detection in the existing of interferences.

## 4. Conclusions

A novel paper-based screen-printed ionic liquid/graphene modified with PB/Ti_3_C_2_T_x_/GOx and Nafion was successfully fabricated for glucose sensing in real plasma samples. The use of ionic liquid/graphene ink to create an electrode on a paper substrate was described for the first time. With the simple modification process, the PB/Ti_3_C_2_T_x_/GOx/Nafion SPIL-GE can be applied to manufacture scale in the future. the advantageous properties of MXene such as good in conductivity and catalytic activity synergies with the outstanding properties of PB which can be measured glucose at low potential. This strategy has been applied in glucose determination to avoid the disturbed signal from interference species at high potential and enhance sensitivity. This promising platform can potentially be used for in-field analysis and applied to monitor other reactions that produce H_2_O_2_ as a by-product.

## Figures and Tables

**Table 1:** Result of glucose detection in blood plasma samples based on electrochemical method and automated analyzer (Hexokinase method).

Samples	Paper-Based PB/Ti_3_C_2_T_x_/Gox/Nafion/SPIL-GE (mM ± sd)	Automated Analyzer (Hexokinase Method) (mM)
1	4.46 ± 0.36	4.67
2	8.23 ± 1.14	8.00
3	12.64 ± 1.04	12.61
4	4.31 ± 0.51	4.28
5	7.07 ± 0.63	7.67
6	12.36 ± 0.59	12.50
7	6.40 ± 1.46	6.22
8	9.92 ± 1.17	9.61
9	9.57 ± 0.38	10.22
10	10.32 ± 0.59	10.06

**Table 2:** Comparative performance of this as-prepared sensor and some others for the determination of glucose.

Sensor *	Technique	LOD	Analytical Range	Reference
GOx/AuNP/PANI/rGO/NH_2_-MWCNTs modified SPCE	Chronoamperometry	0.064 mM	1–10 mM	[35]
GA@PB/SPCE GA@PB/SPCE	Chronoamperometry	0.15 mM	0.5–6 mM	[36]
p-taurine/GOx/Nf-modified GCE	Differential pulse voltammetry	0.06 mM	0.9–15 mM	[37]
Nafion/GOx/SiIONPs/SPCEs	Chronoamperometry	0.22 mM	Up to 3 mM	[38]
The paper-based PB/Ti_3_C_2_T_x_/GOx/Nafion/SPIL-GE	Chronoamperometry	0.024 mM	0.08–15 mM	Present work

* GOx/AuNP/PANI/rGO/NH2-MWCNTs modified SPCE = Glucose oxidase amine-terminated multiwall carbon nanotubes/reduced graphene oxide/polyaniline/gold nanoparticles modified screen-printed carbon electrode (SPCE), GA@PB/SPCE GA@PB/SPCE = Porous graphene aerogel/prussian blue modified screen-printed electrode, p-taurine/GOx/Nf-modified GCE = glucose oxidase at poly(taurine) modified glassy carbon electrode, Nafion/GOx/SiIONPs/SPCEs = Nafion/glucose oxidase/silica-encapsulated iron oxide nanoparticles modified screen-printed carbon electrode.

**Table 3:** Effects of interferences on the paper-based PB/Ti_3_C_2_T_x_/GOx/Nafion/SPIL-GE.

Test Substances	% Recovery
Glucose 5 mM	100
Glucose 5 mM + Uric Acid (0.1 mM)	91.1
Glucose 5 mM + Ascorbic Acid (0.1 mM)	97.3
Glucose 5 mM + Hemoglobin (0.06 mM)	93.2

## Data Availability

Not applicable.

## Supplementary Materials

The following supporting information can be downloaded at: https://www.mdpi.com/article/10.3390/bios12100852/s1, Figure S1: The picture of the physical device for the electrochemical characterization using a portable Palm-Sens4 (a) and the electrochemical detection of plasma glucose using USB-C Sensit Smart connected to smartphone device (b). Figure S2: The resulting mixture of Ti_3_C_2_T_x_ after the first washing cycle (a), the same Ti_3_C_2_T_x_ sediment after washing with deionized water until the pH of supernatant was 5–6, showing the top black Ti_3_C_2_T_x_ layer and grey layer of Ti_3_C_2_T_x_/Ti_3_AlC_2_ mixture at the bottom of the centrifuge tube (b), and the resulting Ti_3_C_2_T_x_ film (c). Figure S3: Cyclic voltammetry of bare SPIL-GE, modified Ti_3_C_2_T_x_/SPIL-GE, and PB/Ti_3_C_2_T_x_/SPIL-GE with 0.1 M KCl (Inset: CV of bare SPIL-GE).
