# Supplementary material for: Paper-Based Screen-Printed Ionic-Liquid/Graphene Electrode Integrated with Prussian Blue/MXene Nanocomposites Enabled Electrochemical Detection for Glucose Sensing
Source: Biosensors (Basel). 2022 Oct 9;12(10):852. doi: 10.3390/bios12100852 (PMC9599729; doi:10.3390/bios12100852)
Supplement: Supplementary file 1 [file biosensors-12-00852-s001.zip › biosensors-1890910-supplementary.pdf]

Paper-Based Screen-Printed Ionic-Liquid/Graphene Electrode Integrated with Prussian Blue/MXene Nanocomposites Enabled Electrochemical Detection for Glucose Sensing

Wisanu Niamsi [1], Nutcha Larpant [2], Pramod K. Kalambate [2], Vitsarut Primpray [3], Chanpen Karuwan [3], Nadnudda Rodthongkum [4,5] and Wanida Laiwattanapaisal [2,*]

[1] Graduate Program in Clinical Biochemistry and Molecular Medicine, Faculty of Allied Health Sciences, Chulalongkorn University, Bangkok 10330, Thailand
[2] Biosensors and Bioanalytical Technology for Cells and Innovative Testing Device Research Unit, Department of Clinical Chemistry, Faculty of Allied Health Sciences, Chulalongkorn University, Bangkok 10330, Thailand
[3] Graphene Sensor Laboratory (GPL), Graphene and Printed Electronics for Dual-Use Applications Research Division (GPERD), National Security and Dual-Use Technology Center (NSD), National Science and Technology Development Agency (NSTDA), Phahonyothin Road, Khlong Nueng, Khlong Luang, Pathum Thani 12120, Thailand
[4] Metallurgy and Materials Science Research Institute, Chulalongkorn University, Bangkok 10330, Thailand
[5] Center of Excellence in Responsive Wearable Materials, Chulalongkorn University, Bangkok 10330, Thailand
* Correspondence: wanida.l@chula.ac.th

Supporting Information

This supplementary section provides the actual picture of the physical device for electrochemical characterization and the analytical performance of glucose determination using portable potentiostat device, the additional description of $Ti_3C_2T_x$ synthesis and the electrochemical characterization using modified electrodes in 0.1 M KCl by CV to verify the redox signal of PB modified electrode.

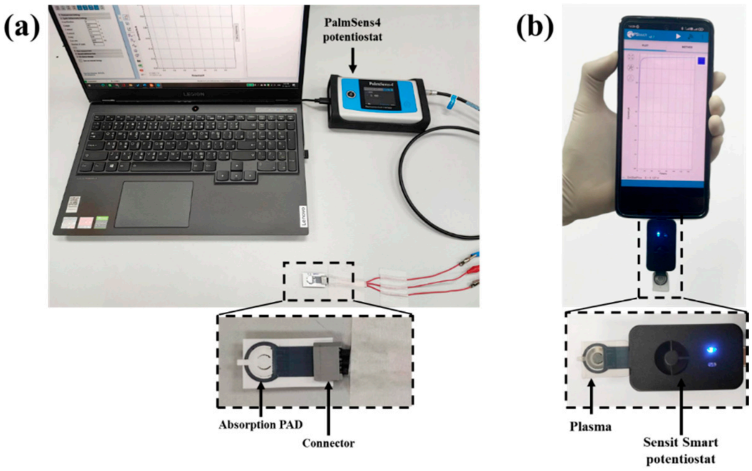

Figure S1. The picture of the physical device for the electrochemical characterization using a portable PalmSens4 (a), the electrochemical detection of plasma glucose using USB-C Sensit Smart connected to smartphone device (b).

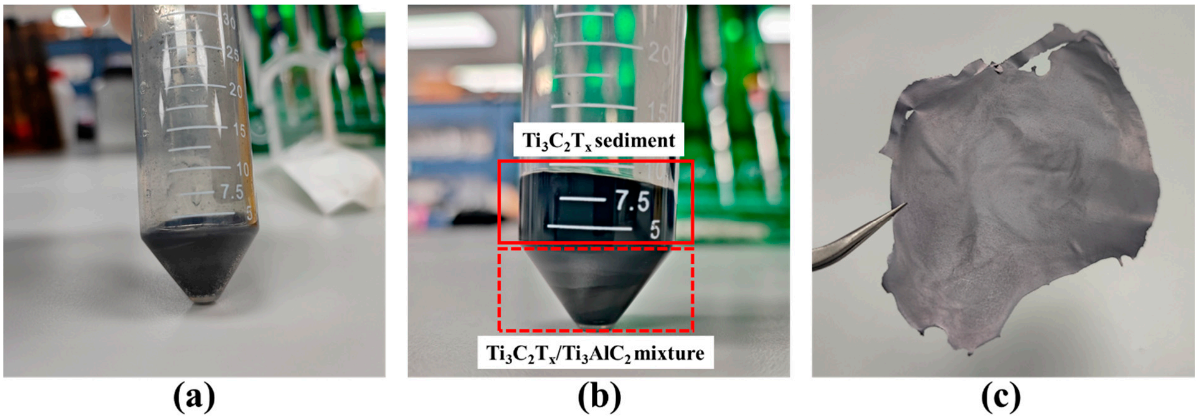

Figure S2. The resulting mixture of $Ti_3C_2T_x$ after the first washing cycle (a), the same $Ti_3C_2T_x$ sediment after washing with deionized water until the pH of supernatant was 5-6, showing the top black $Ti_3C_2T_x$ layer and grey layer of $Ti_3C_2T_x/Ti_3AlC_2$ mixture at the bottom of the centrifuge tube (b), and the resulting $Ti_3C_2T_x$ film (c).

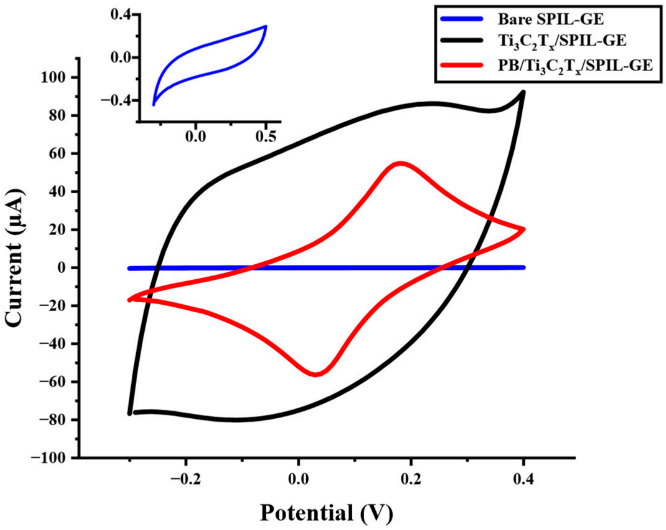

Figure S3. Cyclic voltammetry of bare SPIL-GE, modified $Ti_3C_2T_x$/SPIL-GE, and PB/$Ti_3C_2T_x$/SPIL-GE with 0.1 M KCl (Inset : CV of bare SPIL-GE)
